# Biodefensive Based on *Piper nigrum* Essential Oil for Controlling of *Anopheles aquasalis* Larvae: Influence of Temperature (35 °C) and Preservatives

**DOI:** 10.3390/biom12111711

**Published:** 2022-11-18

**Authors:** Ayná Caroline Marcião Vieira, Sidney Gomes Azevedo, Ramon Andrade Linhares, Silvia Cássia Brandão Justiniano, Grafe Oliveira Pontes, Alessandra Ramos Lima, Pedro Henrique Campelo, Jaqueline de Araújo Bezerra, Camila da Costa Pinto, Henrique Duarte da Fonseca Filho, Robert Saraiva Matos, Ştefan Ţălu, Vanderlei Salvador Bagnato, Natalia Mayumi Inada, Edgar Aparecido Sanches

**Affiliations:** 1 Laboratory of Nanostructured Polymers (NANOPOL), Federal University of Amazonas (UFAM), Manaus 69067-005, Brazil; 2 Graduate Program in Materials Science and Engineering (PPGCEM), Federal University of Amazonas (UFAM), Manaus 69067-005, Brazil; 3 Tropical Medicine Foundation Doctor Heitor Vieira Dourado (FMT-HVD), Manaus 69040-000, Brazil; 4 São Carlos Institute of Physics (IFSC), University of São Paulo (USP), São Carlos 13563-120, Brazil; 5 Department of Food Technology, Federal University of Viçosa (UFV), Viçosa 36570-900, Brazil; 6 Federal Institute of Education, Science and Technology of Amazonas (IFAM), Manaus 69020-120, Brazil; 7 Laboratory of Nanomaterials Synthesis and Nanoscopy (LSNN), Federal University of Amazonas (UFAM), Manaus 69067-005, Brazil; 8 Amazonian Materials Group, Federal University of Amapá (UNIFAP), Macapá 68903-419, Brazil; 9 The Directorate of Research, Development and Innovation Management (DMCDI), Technical University of Cluj-Napoca, 15 Constantin Daicoviciu St., 400020 Cluj-Napoca, Romania; 10 Hagler Institute for Advanced Studies, Texas A&M University, College Station, TX 77843-3572, USA

**Keywords:** biodefensive, encapsulation, essential oil, *Piper nigrum*, *Anopheles aquasalis*

## Abstract

Considerable efforts have been spent on the development of biodefensives based on the encapsulation of essential oils for controlling of urban pests from their larval stage, especially as anopheline controlling agents. The larval source management of *Anopheles aquasalis* is important for malaria prevention. For this reason, this research proposes larvicidal biodefensives based on polymeric particles loaded with *Piper nigrum* essential oil, considering the influence of temperature (35 °C) and preservatives on the formulation stability. The biodefensive containing the preservative phenoxyethanol/methylisothiazolinone (PNE) resulted in 5 months of shelf-life storage with an Encapsulation Efficiency (EE%) of essential oil of 70%. The biodefensive PNE (containing 500 µg.mL^−1^ of encapsulated essential oil) presented a polydisperse particle size distribution, ranging from D_10_ = (127 ± 10) nm to D_90_ = (472 ± 78) nm and a particle mean size of (236 ± 34) nm. The AFM images revealed a spherical morphology with an external surface almost regular and smooth. The controlled release of the essential oil was evaluated up to 72 h according to the Korsmeyer-Peppas mathematical model, confirming the anomalous transport (*n* = 0.64 in pH = 3 and pH = 10, and *n* = 0.65 in pH = 7). The total larvae mortality on the *in loco* bioassays was almost reached (92%) after 24 h. However, according to the *in vitro* bioassays applying the *in natura* essential oil alone, the concentration of 454 μg.mL^−1^ resulted on the mortality of 70% of the larvae after 24 h. For this reason, the highest efficiency of the biodefensive PNE may be related to the encapsulation of essential oil, delivering the loaded particles more efficiently inside the larvae. From this perspective, the present study shows that a formulation based on *P. nigrum* essential oil may be taken into account in the integrated management of disease vector mosquitoes.

## 1. Introduction

Mosquitoes are a major health problem today because they transmit diseases such as malaria, dengue fever, yellow fever, and the Zika virus, which impact 700 million people each year and kill over one million people [1]. The use of essential oils as biodefensives has been extensively reported, especially as anopheline controlling agents [2,3,4]. Brazil has the fourth highest prevalence of malaria in Americas, and most cases occur in the Amazon region [5]. Presently, strategies to control mosquito-borne diseases are limited to mosquito population reduction and, in case of malaria, to drugs. However, no drugs are available to treat viral diseases [1]. For this reason, vector controlling is important for malaria prevention; the larval source management targets the immature, aquatic stages of mosquitos and reduces the abundance of adult vectors [6]. Unfortunately, the excessive and inappropriate application of chemical pesticides have gradually resulted in food and environmental contamination, as well as in resistant biotypes [7].

The numerous advantages of biodefensives have stimulated a growing interest in the development of novel formulations based on bioactive molecules such as plant secondary metabolites [8,9]. For this reason, essential oils have been extensively investigated as alternative controlling agent due to their important fungicide, insecticide and larvicidal properties [8,10,11,12]. The effectiveness of the essential oil from black pepper (*Piper nigrum* L.; Piperaceae), known in Brazil as “pimenta-do-reino”, was evaluated herein against *Anopheles aquasalis* (Diptera: Culicidae) larvae. The larvicidal effects of black pepper and piperine against insecticide resistant and susceptible strains of *Anopheles* malaria vector mosquitoes were previously reported [13], pointing to a viable possibility of encapsulation of this essential oil for the development of a biodefensive.

The encapsulation of essential oils in polymeric particles has resulted in several alternative biodefensives that present low toxicity and biodegradability [7,11,14,15]. The encapsulation technique has become highly integrated in the development of biodefensives, since colloidal systems are prominent carriers of bioactive molecules [16,17]. However, the knowledge about both particle core and carrier is fundamental for the development of new materials with desired releasing mechanism. Gelatin and poly-*ε*-caprolactone (PCL) have been applied successfully as carriers because they are FDA approved, biodegradable and non-toxic, in addition to presenting good encapsulating efficiency [9,18,19,20].

Gelatin/PCL-based particles loaded with essential oil from *P. nigrum* were successfully prepared herein and maintained at 35 °C. Different preservatives were considered to verify their influence on the formulation stability. High Encapsulation Efficiency (EE%) is not a single parameter to guarantee the formulation stability over time [18,21,22]. The use of preservatives in colloidal systems is an effective alternative to increase their shelf-life [23], especially by delaying the exposure of bioactive compounds into the medium as a result of the particles destabilization/rupture [21]. For this reason, several parameters are simultaneously correlated to the stability of colloidal systems, such as pH, electrical conductivity, EE% and turbidity [18]. The biodegradability of gelatin/PCL-based particles in colloidal systems is a natural process, so the development of preservative-containing formulation allows the increase of their shelf-life [21,23].

Biodefensives containing different preservatives were developed and fully characterized. The stability of the biodefensives was evaluated over time according to physical parameters such as EE%, pH, electrical conductivity, and turbidity. Nanoparticle Tracking Analysis (NTA) and Zeta potential were useful for particle size distribution and surface charge measurements, respectively. Atomic Force Microscopy (AFM) allowed the evaluation of the particle’s morphology and external surface roughness. The stability evaluation of the developed systems was performed at (35 ± 2) °C based on established protocols [22], considering constant handling and shelf-life tests. The controlled release of the essential oil was evaluated under different pH (3, 7 and 10), and the experimental curves were fitted according to mathematical models [24,25]. Finally, the biodefensive PNE was tested on *in-vitro*/*in-loco* bioassays against *A. aquasalis* larvae to access its larvicidal effectiveness.

## 2. Materials and Methods

### 2.1. Essential Oil Extraction

*P. nigrum* fruits (SISGEN Authorization A26CD5E) were purchased at the Adolpho Lisboa market in Manaus/AM. For essential oil extraction, 300 g of dried fruits were subjected to hydrodistillation using a modified Clevenger-type apparatus for 3 h at (97 ± 3) °C. The essential oil was dried over anhydrous sodium sulphate and stored at −18 °C. The essential oil yield (w/v) was based on the ratio between the extracted oil volume and the seed mass. The relative density of the essential oil was estimated at 20 °C [26,27]. The refraction index of the raw essential oil was estimated at 20 °C using an Atago Master-T series refractometer (Ribeirão Preto, São Paulo, Brazil).

### 2.2. Gelatin/PCL-Based Particles and Encapsulation Efficiency

Gelatin/PCL-based particles loaded with *P. nigrum* essential oil were synthesized according to previous report [9,21] with modifications. The same methodology was performed without essential oil (in solution II) to obtain the colloidal system containing unloaded particles. The preservatives (i) citric acid (0.1%; PCA), (ii) phenoxyethanol/methylisothiazolinone (0.01%; PNE), (iii) methylisothiazolinone (0.0015%; PMI), (iv) sodium benzoate (0.1%; PBS), (v) thymol (0.15%; PTH) or (vi) ethylenediaminetetraacetic acid (0.1%; PED) were added in the formulations. Moreover, a formulation containing loaded particles without preservatives (labeled as PNP), as well as the formulation containing unloaded particles without preservatives (labeled as PUN) were also synthesized. All formulations were maintained at (35 ± 2) °C.

EE% was evaluated using UV-VIS spectroscopy [28]. A known concentration of essential oil in ethanol was scanned in the range of 190–400 nm on a Epoch2 Microplate Reader Biotek (Agilent, Santa Clara, CA, USA). A sharp peak was noticed at 278 nm. From the calibration curve (Y = 0.0245x + 0.0679; R^2^ = 0.99885), the unknown concentration of essential oil was obtained by possessing the absorbance values. Particles were separated by centrifugation (20.000 rpm), and the supernatant absorbance was used to determine the percentage of free essential oil. Then, EE% was calculated using the formula: EE% = (amount of encapsulated essential oil/ total amount of essential oil used in the formulation) × 100. Experiments were carried out in triplicate.

### 2.3. Stability Evaluation

The stability evaluation of the developed biodefensives was based on established protocols [22] with modifications.

All systems were previously submitted to 3.000 rpm for 30 min. Then, stability tests were performed as follows:

(i). Stability under constant handling: Biodefensives containing essential oil and preservatives (PCA, PNE, PMI, PTI, PED and PBS) were stored in transparent vials, and maintained at (35 ± 2) °C. Vials were opened (and consequently exposed to the laboratory environmental conditions such as air contact, light, and temperature variation) at pre-established time intervals (1–3 days) for 30 days. Physical parameters (pH, electrical conductivity, EE% and turbidity) were measured each time the vials were opened. Then, the more stable biodefensives were evaluated again for 120 days. All measurements were performed in triplicate.
(ii). Shelf-life test: The biodefensives selected in the stability test under constant handling at (35 ± 2) °C were submitted to the shelf-life test at (35 ± 2) °C. Biodefensives were stored in sealed vials until reach EE% equal to or less than 70%. Vials were opened every 30 days. Physical parameters (pH, electrical conductivity, EE% and turbidity) were measured each time the vials were opened. All measurements were performed in triplicate.

### 2.4. Essential Oil Release

Essential oil release evaluation was carried out *in vitro* at different pH (3, 5 and 7). Biodefensive (15 mL) was inserted in dialysis tubing cellulose membrane, suspended in water (85 mL) and maintained under continuous magnetic stirring (100 rpm). An aliquot (3 mL) was withdrawn at regular time intervals (up to 120 h), and the absorbance values were obtained at 278 nm using an Epoch2 Microplate Reader Biotek spectrophotometer (Agilent, Santa Clara, CA, USA). The amount of released essential oil was calculated from a standard curve [29]. The cumulative release (%) of essential oil was obtained from the following equation: Cumulative release (%) = (amount of essential oil released after time t/total amount of encapsulated essential oil) × 100. Experiments were carried out in triplicate. The mechanism of essential oil release was evaluated according to the Korsmeyer-Peppa’s [25] mathematical model.

### 2.5. Zeta Potential

Zeta potential values (in mV) were measured on a Zetasizer Nano ZS90 (Malvern Instruments, Malvern, UK). Samples (unloaded particles and loaded particles containing an absolute concentration of 500 μg.mL^−1^ of essential oil) were analyzed at 25 °C. Measurements were performed in triplicate.

### 2.6. Nanoparticle Tracking Analysis (NTA)

NTA measurements were performed on a NanoSight NS300 device (Malvern Instruments, Malvern, UK), equipped with a green laser type. Samples were diluted in MilliQ water (1:100 v/v). A standard operating procedure was created using 749 frames for 30 s. Particle size was corrected for viscosity of the continuous phase (water, 0.9 cP). Measurements were performed in triplicate. The evaluation of the Particle Size Distribution (PSD) was performed with the parameters Mean, Mode, SD, D10, D50 (Median) and D90 which indicate, respectively, the average, most frequent particle class size, standard deviation of the distribution, and the 10%, 50% and 90% percentiles of the analyzed particles.

### 2.7. Atomic Force Microscopy (AFM)

Formulation containing unloaded (PUN) and loaded (PNE) particles (1 µL) were dripped on a glass slide and allowed to dry. Measurements were performed in air at room temperature (296 ± 1 K) and (40 ± 1)% R.H. on an Innova equipment (Bruker, Billerica, MA, USA) operated in tapping mode and equipped with a silicon tip and Al coated cantilever with a spring constant of 42 N/m (Tap190AL-G from Budget Sensors^TM^, Sofia, Bulgaria). Scans were performed using (10 × 10) µm^2^ with (256 × 256) pixels at a scan rate of 1 Hz.

### 2.8. Thermogravimetry/Derivative Thermogravimetry (TG/dTG) and Differential Scanning Calorimetry (DSC)

TG/*d*TG and DSC techniques were performed on a SDT Q600 TA Instrument (Thermal Analysis Instruments, New Castle, DE, USA). Measurements were carried out using approximately 10 mg of samples in alumina crucibles, N_2_ atmosphere (flow of 30 mL/min) at heating rate of 10 °C/min, from 25 °C to 600 °C.

### 2.9. Larvicidal Bioassays

Larvicidal bioassays were carried out based on previous reports [8,30] with marginal modifications under *in vitro* ((27 ± 2) °C; (75 ± 5)% R.H.) and *in loco* ((29 ± 1) °C; (65–72)% R.H.) conditions. Groups of 10 *A. aquasalis* larvae (3rd larval stage) were placed in vials containing 20 mL of larvae breeding water. Essential oil was solubilized in Tween 80^®^ (0.03%) to prepare concentrations of 125, 250, 375, 454 and 625 µg.mL^−1^. Then, 10 mL was added to the previous vials containing larvae. The essential oil effectiveness was evaluated after 24 h, 48 h and 72 h. Tween 80^®^ (0.03%) and Bti (1 mg/mL^−1^) were used as negative and positive control, respectively. Data were analyzed using the POLO PC^®^ program [31]. Then, the LD_50_ (Lethal Dosage that kills 50% of the exposed larvae), LD_90_ (Lethal Dosage that kills 90% of the exposed larvae), LCL (Lower Confidence Limit) and UCL (Upper Confidence Limit) were estimated with fiducial limit of 95% [32]. Experiments were performed in triplicate.

Larvicidal bioassays were also conducted to evaluate the biodefensive efficiency. Groups of 20 larvae (total of 600 larvae; 3rd larval stage) were placed in vials containing 20 mL of breeding water. Biodefensive (10 mL) was diluted in water to prepare concentrations of 100, 200, 400 and 500 µg.mL^−1^ of encapsulated essential oil. Unloaded particles (10 mL) and thymol (3 μg.mL^−1^) were used as negative and positive control, respectively. The biodefensive effectiveness was evaluated after 24 h, 48 h and 72 h. Experiments were carried out in triplicate.

### 2.10. Statistical Analysis

Data were expressed as mean ± standard deviation for three replications. The multiple data comparison was evaluated by ANOVA. The LSD (least significance difference) intervals (*p*-value < 0.05) were estimated. The Duncan’s test at 95% of confidence level was applied. Statistical analyses were carried out using the *R*-software package (version 3.6.0) (Vienna, Austria).

## 3. Results and Discussion

### 3.1. Stability under Constant Handling at (35 ± 2) °C

**Figure 1:**
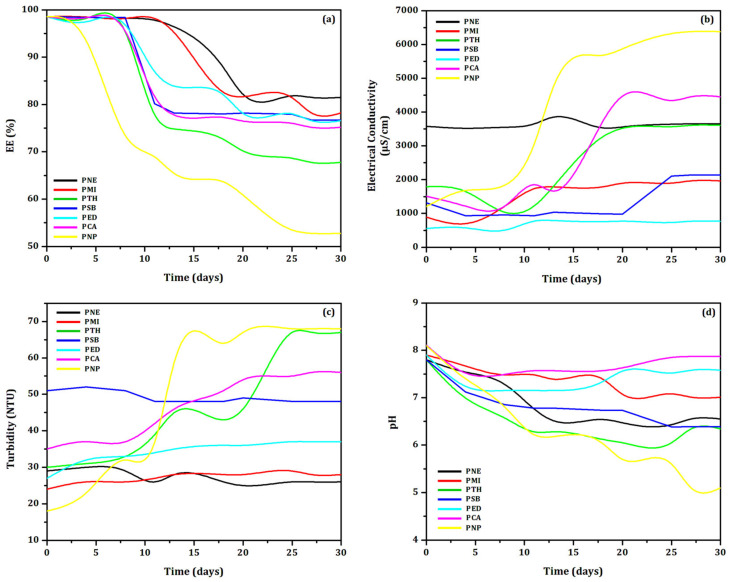
Evaluation of (**a**) Encapsulation Efficiency (EE%), (**b**) electrical conductivity, (**c**) turbidity and (**d**) pH of all developed biodefensives (PNE, PMI, PTH, PSB, PED, PCA and PNP) for 30 days.

The stability of the biodefensives was evaluated under constant handling at (35 ± 2) °C according to the physical parameters EE%, electrical conductivity, turbidity, and pH for 30 days at regular time intervals. Results are shown in Figure 1.

The most stable systems were determined mainly by the evaluation of EE%, which are considered significant above 70% [33]. Figure 1a shows the decrease of the EE% as a function of time in all systems, which presented initial EE = (98 ± 2)% (except the unloaded particles system, PNP). The high initial EE% showed the success of the developed loaded particles. However, the evaluation of the EE% is not a single parameter to guarantee the stability of the formulation over time [18,21].

The systems PTH and PNP presented considerably reduced EE% after 30 days. The results also showed that all biodefensives containing preservatives showed EE% values higher than that of PNP, as expected. The biodefensives PED, PSB and PCA reached EE∼75% after 30 days. The biodefensives that clearly showed greater stability at (35 ± 2) °C as a function of EE% were PNE and PMI, which reached EE = 81% and EE = 78%, respectively, after 30 days. Considering that PNP reached EE = 70% after 10 days, we can suggest that all preservatives increased the stability of biodefensives in at least 20 days.

The results of the stability evaluation as a function of electrical conductivity are shown in Figure 1b. The biodefensives PTH, PCA and PNP presented a significant increase of electrical conductivity after 14 days. This result corroborates the EE% evaluation, indicating a progressive destabilization. The values ranged from (1.786 ± 5) µS.cm^−1^ to (3.593 ± 5) µS.cm^−1^ in the biodefensive PTH; from (1.481 ± 4) µS.cm^−1^ to (4.468 ± 4) µS.cm^−1^ in PCA, and from (1.118 ± 5) µS.cm^−1^ to (6.376 ± 5) µS.cm^−1^ in PNP. The increase of the electrical conductivity may be related to the exposure of essential oil into the medium [18,34], probably due to particles rupture [23]. However, other factors can be considered, such as, in this case, the temperature of storage (35 ± 2) °C [35]. The electrical conductivity values also suggest modification of the isoelectric point [36]. The biodefensives PED, PNE and PMI presented marginal changes of electrical conductivity, corroborating the EE% results. The biodefensive PED showed electrical conductivity between (566 ± 5) µS.cm^−1^ and (756 ± 5) µS.cm^−1^ after 30 days; the electrical conductivity of the biodefensive PNE increased from (3.579 ± 5) µS.cm^−1^ to (3.642 ± 5) µS.cm^−1^, and from (859 ± 5) µS.cm^−1^ to (1.952 ± 5) µS.cm^−1^ in PMI after 30 days. Changes of electrical conductivity in colloidal systems may be related to particle rupture, while the decrease is associated to particle aggregation [22]. Our results pointed to the increase of electrical conductivity due to the exposure of the essential oil into the medium. This result may be also related with the observed turbidity variation.

Figure 1c shows the turbidity of the biodefensives as a function of time. At t = 0, all biodefensives presented turbidity from (18 ± 1) NTU to (52 ± 1) NTU. This fact shows that the addition of preservatives influenced on the initial turbidity values. The highest turbidity variation was observed in the biodefensives PTH, PCA and PNP. Marginal changes of turbidity were observed in the biodefensives PNE and PMI. Values ranged from (29 ± 3) NTU to (26 ± 3) NTU in PNE, and from (24 ± 2) NTU to (28 ± 3) NTU in PMI after 30 days. The turbidity variation corroborates the EE% and electrical conductivity results: the turbidity values increased as the biodefensives were gradually destabilized, accompanied by a decrease of EE% and increase of electrical conductivity [22].

The pH variation as a function of time is shown in Figure 1d. According to the Zeta potential analysis [9], all biodefensives were initially adjusted to pH = 8 since the isoelectric point was found at pH = 4 [9]. The pH values of all biodefensives decreased considerably in the first 5 days. Marginal changes of pH were observed in PNE and PMI; values decreased to (6.50 ± 0.03) and (7.00 ± 0.03), respectively, after 30 days. However, significant modification was observed in the biodefensives PTH (final pH = (6.30 ± 0.03)) and PNP (final pH = (5.0 ± 0.03)). The decrease of pH may be explained by the exposure of essential oil into the medium (as a consequence of the particles rupture—suggestion based on the results of EE%, electrical conductivity and turbidity) [37]. However, the decrease of pH over time in colloidal systems may also be associated with the degradation of the materials from formulation, inducing acidification [38]. This result can also indicate polymeric degradation due to the ionization of carboxylic groups and hydrolysis [39]. The possibility of gelatin degradation by reactions with free carboxylic groups can also be suggested [40].

**Figure 2:**
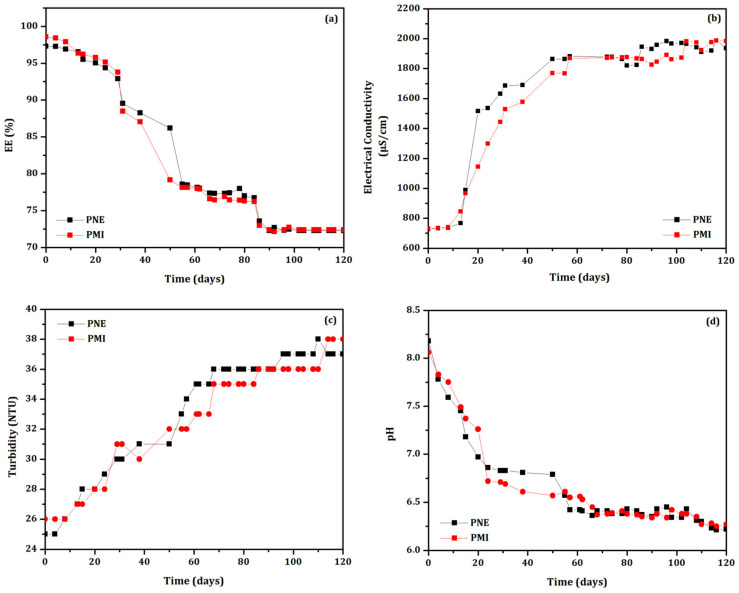
Evaluation of (**a**) EE%, (**b**) electrical conductivity, (**c**) turbidity, and (**d**) pH of the biodefensives PNE (black curves) and PMI (red curves) maintained under constant handling at (35 ± 2) °C for 30 days.

Only the biodefensives PNE and PMI maintained their pH, electrical conductivity, and turbidity, as well as an EE% higher than 70% as a function of time. Then, both systems were further evaluated under constant handling at (35 ± 2) °C for 120 days, as shown in Figure 2. The biodefensives PNE and PMI presented similar behavior of EE% as a function of time: the values were considerably reduced after 55 days, reaching EE = 78%. Then, the EE% reduced marginally up to 120 days, reaching 73%. The pH, electrical conductivity and turbidity parameters followed the EE% changes and were influenced by the exposure of essential oil into the medium probably due to the particle’s destabilization. After 120 days of evaluation, the final values of pH, electrical conductivity and turbidity were, respectively, 6.2, 1971 µS.cm^−1^ and 37 NTU.

Few reports have evaluated the variation of physical parameters as a function of time [18,21,41]. Poly-*ɛ*-caprolactone (PCL) nanoparticles were loaded with essential oil (500 μg.mL^−1^) from *Lippia alba* to develop a larvicidal controlled-release biodefensive containing preservatives [18]. The authors reported that the biodefensive without preservative presented considerable decreasing of EE% after 30 days (reaching 66%). On the other hand, the addition of the preservatives phenoxyethanol/isotialzoni-3-one and sodium benzoate increased the formulation stability: the EE% for both formulations presented similar behavior over time, reaching 70% after 50 days. Another recent study [21] reported the stability of a biodefensive based on polymeric particles containing *P. nigrum* essential oil maintained at 25 °C. The preservative phenoxyethanol/isotialzoni-3-one allowed an enhanced stability of approximately 120 and 210 days, respectively, under constant handling and shelf-life tests.

### 3.2. Shelf-Life Storage at (35 ± 2) °C 

**Figure 3:**
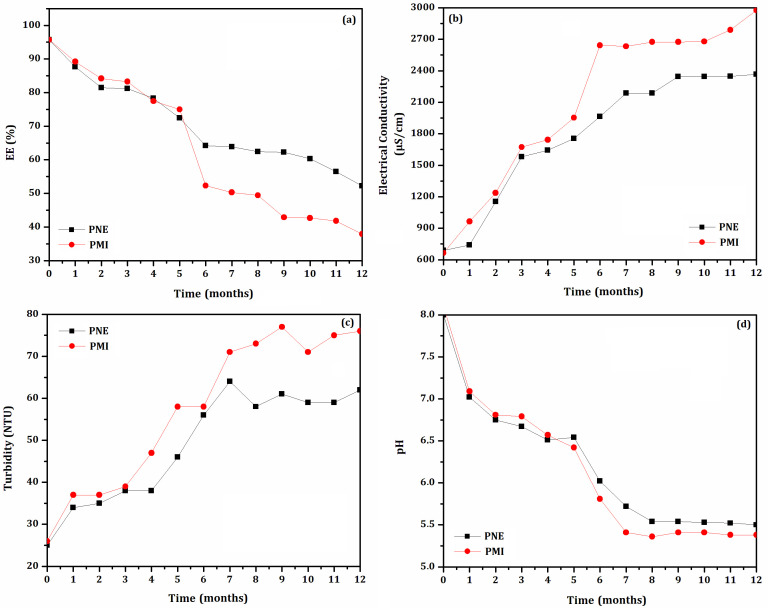
Shelf-life storage evaluation (12 months) of the biodefensives PNE and PMI based on (**a**) Encapsulation Efficiency (EE%), (**b**) electrical conductivity, (**c**) turbidity and (**d**) pH.

The stability of the biodefensives PNE and PMI was evaluated at (35 ± 2) °C for 12 months. Results are shown in Figure 3. Monthly analyzations showed similar results as those found in constant handling evaluations. However, the shelf-life evaluation presented higher values of EE%. Both biodefensives maintained their physical properties for up to 12 months. However, particulate material was observed at the bottom of the vials after 5 months. This flocculation can be related to the increase of turbidity and decrease of EE%, as previously observed.

The biodefensive PNE presented higher EE% than that of PMI after 12 months. A value of EE = 70% [33] was reached after 5 months for both biodefensives. Then, the EE% of the biodefensives PNE and PMI decreased considerably, reaching 65% and 38%, respectively, after 12 months. The biodefensive PNE presented improved stability in all evaluated parameters, including pH and EE%, as well as lower electrical conductivity and turbidity.

The biodefensive PNE presented reduced shelf-life at (35 ± 2) °C (5 months) when compared to the evaluation performed at (25 ± 2) °C (7 months) [21]. This result is possibly related to the influence of temperature on the biodefensive stability. However, our results also showed that the preservative phenoxyethanol/methylisothiazolinone positively influenced PNE stability. Phenoxyethanol has a large spectrum of antimicrobial activity, and has been used at concentrations below 1% as a preservative in cosmetic products and fixative in perfumes [42]. It is effective against various Gram-negative and Gram-positive bacteria, as well as against yeasts, and has only a weak inhibitory effect on resident skin flora [43]. Furthermore, methylisothiazolinone has been used in industrial products since the early 2000s and, in 2005, it was allowed to be used as a preservative in cosmetic products [44] at a maximum concentration of 0.01% [45]. However, the preservative action was more efficient on the tested formulation performed herein when combining phenoxyethanol and methylisothiazolinone. Similar results of stability were also obtained previously [21].

### 3.3. Zeta Potential and Particle Size Distribution

The Zeta potential is an important physicochemical parameter that can be correlated with the stability of colloidal systems [46]. Higher values (in module) of Zeta potential are related to repulsion and reduction of aggregation/agglomeration [47]. There was a tendency for both PNE and PMI biodefensives to maintain their isoelectric points (ζ~0) from pH = 2 to pH = 4 [9]. However, between pH = 5 and pH = 8, both biodefensives presented increased surface charge, which reached highest value (in module) in pH = 8 [9]: PNE and PMI presented Zeta potential values of −30.6 mV and −18.5 mV, respectively.

Similar values of Zeta potential were found previously [48]. Another indicative of destabilization by charges was observed in the biodefensive PMI: the conductivity (0.4250 mS.cm^−1^) was higher than that of PNE (0.0127 mS.cm^−1^), suggesting a greater number of free charges in the medium. The electrical conductivity of the biodefensives is related to pH, which can change drastically when the formulation shifts from the isoelectric point [18]. For this reason, particles destabilization is suggested in the biodefensive PMI. Thus, only the biodefensive PNE was selected for further analyzes, as well as for the larvicidal bioassays.

**Figure 4:**
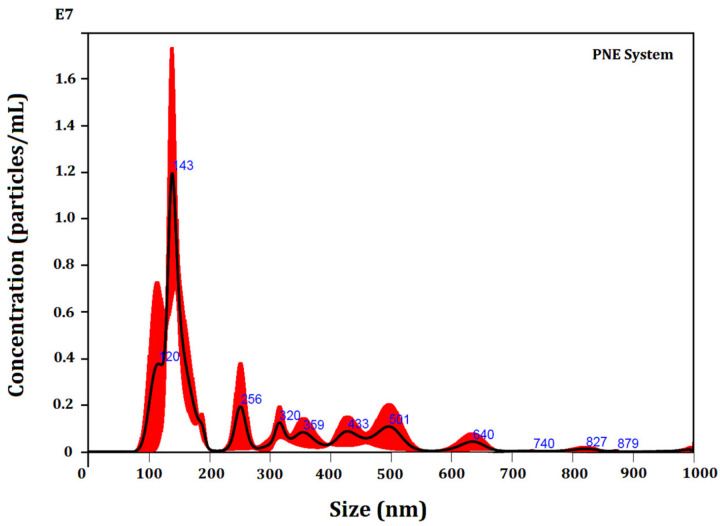
NTA particle size distribution analysis of the biodefensive PNE. Representative histogram of the average size distribution (black line) from three measurements of a single sample. Red areas specify the standard deviation (SD) between measurements, and blue numbers indicate the maxima of individual peaks.

The biodefensive PNE was also characterized according to its particle’s population by NTA (Table 1 and Figure 4), revealing a polydisperse particle size distribution, ranging from D_10_ = (127 ± 10) nm to D_90_ = (472 ± 78) nm. The particles mean size was found as (236 ± 34) nm, which is similar to previous study [21]. The mode parameter shows the particle size (or size range) most commonly found in the population distribution, and this is helpful to describe the midpoint for non-symmetric distributions [49]. The value that best represents the biodefensive PNE was (136 ± 10) nm. 

**Figure 5:**
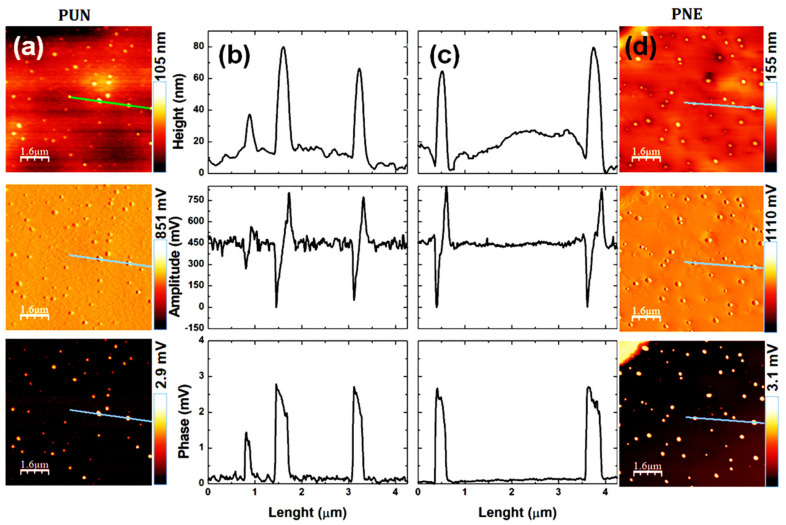
AFM images evaluation based on (**a**) topography, (**b**) amplitude (error signal), (**c**) phase images and (**d**) graphic profiles of the highlighted line of the biodefensives PUN and PNE.

The AFM technique has the advantage of imaging almost any type of material surface because it can identify large bimodal size distributions [50]. The particle size and shape were estimated using the AFM technique and revealed that both unloaded (PUN) and loaded (PNE) particles presented an almost spherical morphology and are well dispersed, as shown in Figure 5. To avoid particle aggregation, it is required that particles are protected against irreversible contact by van der Waals forces, taking advantage of the protective electrostatic repulsion [51]. Zeta potential revealed that both systems presented considerable external surface charge, as previously discussed.

The AFM images revealed that the external surface of each particle is almost regular and smooth, showing that the polymer carriers acted as a continuous film surrounding the essential oil droplets. The average size of the biodefensives PUN and PNE were found around (190 ± 40) nm and (290 ± 50) nm, respectively. The PDI values were found as 0.17 and 0.22, respectively, for PUN and PNE. Particles presenting average diameter around 300 nm and PDI around 0.2 have uniformity and good stability as a colloidal system [46]. The particle average size was dependent on the essential oil: particles containing encapsulated essential oil presented larger average size. A previous study on gelatin, PCL, and essential oil of *P. nigrum* [21] reported a reduced particle size in loaded particles, which may be related to the influence of temperature on the system stability. Some research also has reported the influence of the encapsulated essential oils on the particles average size, morphology, and surface by using the AFM technique [9,20]. A recent study, based on biodegradable nanoparticle carriers containing different concentrations of essential oil from *Allium sativum* (0, 360, 420 and 460 mg/mL^−1^), reported advanced morphological and fractal aspects of the nanoparticles surface. The authors used AFM topographical images to access statistical parameters, revealing that essential oil encapsulation affected the morphology and surface roughness [19].

Figure 5 also presents the topography, amplitude (error signal), phase images and graphic profiles of the highlighted line of PUN and PNE. The phase image presented a strong contrast on topography. However, the height and phase graphic profile were similar. This fact occurs due to the coupling between topography and phase, since the amplitude (or error) changes in relation to the set-point according to the inclination of the height signal (first derived from topography) during the scan, suffering effects from the inclination of the particle surface. A variation in the phase traces and amplitude was observed; however, it was significantly greater in particles.

The real differences in energy dissipation to the local surface slope due to the surface imperfections is considered and is directly related to the cantilever orientation and phase variation. The contrast observed in the phase images presented a direct relationship with the energy dissipation in the tip-sample system. During scanning, if a lateral component appears in the oscillating motion of the tip, changes in the bias of the cantilever will be observed, producing shear dissipation when the tip contacts the sample surface. These imperfections are due to the viscoelastic properties of the particles: different mechanical properties were observed from the rest of the sample surface. However, using AFM tapping mode it was not possible to identify any differences between the PUN and PNE particles.

### 3.4. TG/dTG and DSC Evaluation

**Figure 6:**
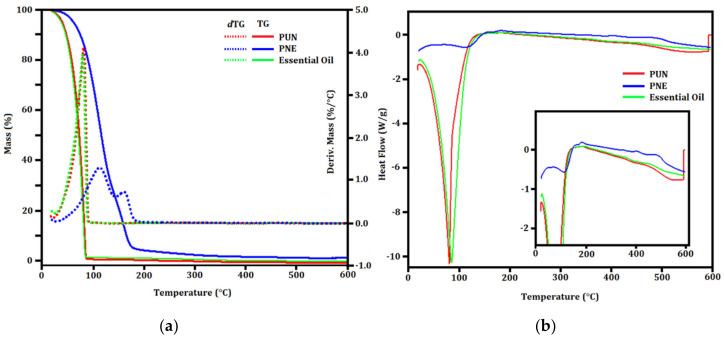
(**a**) TG/*d*TG and (**b**) DSC curves of the biodefensives PUN, NPE and *in natura* essential oil.

TG/*d*TG and DSC evaluation (Figure 6) were carried out to describe the thermal behavior of PUN and PNE. The total degradation of gelatin occurs at approximately 90 °C [52] in a single thermal event. Considering that gelatin represents a higher concentration of the constituents of formulation, it was suggested that for both PUN and PNE, the gelatin degrade simultaneously to the other components. The first peak at approximately 110 °C in the TG curve (Figure 6a) represents the end of the degradation of organic groups, indicating the possible degradation of monoterpenes [53], also suggesting the degradation of high molecular weight organic compounds [54].

A subtle variation in the heat flow was observed in the DSC analysis (Figure 6b) in both PUN and PNE from 362 °C to 478 °C (heat flow variation of 3.35 J.g^−1^), pointing to a residual PCL degradation. Endothermic events were observed in both PUN and PNE, as well as in the *in natura* essential oil. For PNE and PUN systems; the variation of heat flow reached 1614 J.g^−1^. Considering the *in natura* essential oil, an endothermic event with variation of heat flow of 57.92 J.g^−1^ was observed. The TG/*d*TG curve of the raw essential oil showed stability up to ∼80 °C. In sequence, a stage related to the loss of mass due to oil evaporation and decomposition was observed. This result is related to the composition of the *P. nigrum* essential oil, which consists of small organic molecules [21]. As a result of this high thermal stability, this essential oil can be used in encapsulated biodefensives as a natural bioactive for pest controlling in tropical and subtropical countries [8].

### 3.5. Controlled Release

The controlled release of the essential oil was verified by release kinetics. All profiles were analyzed by applying the Korsmeyer-Peppas [25] mathematical model using a simple empirical model [*f = ktn*] [55,56,57]. Results are summarized in Table 2. The mathematical model presented good adjustment to the experimental curves, resulting in R² from 0.93 to 0.96. The kinetic constant *k* is characteristic for a particular system considering structural and geometrical aspects; *n* is the release exponent representing four different mechanisms (Fickian diffusion, anomalous transport, Case-II transport and Super Case-II transport) [58] considering spherical particles, and *t* is the release time. 

The releasing mechanism by Fickian diffusion is the mechanism in which the active diffusion through the particle is exclusively determined by Fickian diffusion. In the case of anomalous transport, the active release is due both to Fickian diffusion and swelling/relaxation of the carrier. The Case-II transport is controlled by the swelling and relaxation of carriers, and it is independent of time. In the Super Case-II transport, the release is ruled by the macromolecular relaxation of the polymeric chains [55].

In general, the *n* value determines the dominant release mechanism. Considering spherical particles, *n* = 0.43 represents a Fickian diffusion; 0.43 ≤ *n* ≤ 0.85 represents an anomalous transport. When *n* = 0.85, the release is ruled by the Case-II transport, and *n* > 0.85 is related to a Super Case-II transport [24]. Our results showed that the essential oil was released from particles by the same mechanism: anomalous transport (*n* = 0.64 in pH = 3 and pH = 10, and *n* = 0.65 in pH = 7).

**Figure 7:**
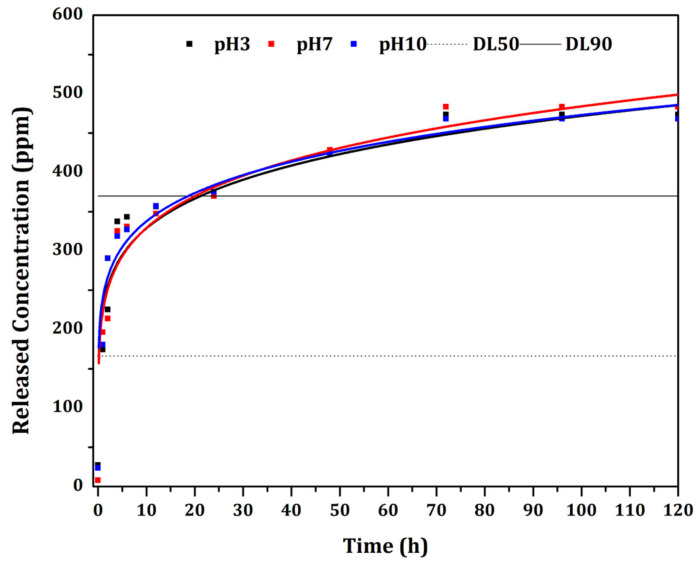
Controlled release curves of the biodefensive PNE at different pH (3, 7 and 10).

The essential oil showed maximum release after 72 h (Figure 7) in all evaluated pH (3, 7 and 10), showing no significant variation. The LD_50_ and LD_90_ values were also considered in Figure 7. The LD_50_ showed values corresponding to those observed in the first 5 h of release. The LD_90_ presented values corresponding to 24 h of essential oil release. According to the release evaluation, concentrations previously estimated for larvicidal application were prepared. Therefore, it is possible to predict the release behavior when subjected to an exposure time of 72 h in the bioassay. The values (in µg.mL^−1^ of the concentration in the medium) after 72 h remained at approximately 460 µg.mL^−1^, indicating the essential oil dispersed in the medium. The value identified in the analysis after 72 h may refer to the maximum concentration of essential oil in the medium.

The anomalous mechanism of controlled release was also reported in a previous study [9] by applying the Korsmeyer-Peppas [25] mathematical model. The authors described the release of *Piper aduncum* and *Piper hispidinervum* essential oils from biodegradable nanoparticles, reaching up to 140 h of release.

### 3.6. Larvicidal Bioassays

A recent study reported by our research group accessed the chromatographic analysis of the essential oil from *P. nigrum*, identifying 29 compounds (95.32%) of the *in natura* essential oil, and *β*-caryophyllene (34.87%) was identified as the major compound [21]. Other studies have reported prominent bioativity of this essential oil, as well as this major compound [59,60,61]. *β*-caryophyllene is present in species such as *Aquilaria crassna*, *Plectranthus barbatus* and *Syzygium aromaticum*, and has been reported to present anticancer, antioxidant, antimicrobial, anaesthetic and anti-inflammatory properties. *Plectranthus barbatus* essential oil and its major chemical constituents (eugenol (31.12%), α-pinene (19.38%) and *β*-caryophyllene (18.42%)) were reported as a safer alternative to mosquito control [62]. Concerning major constituents, the authors reported the effectiveness of this essential oil against *Anopheles subpictus* (LD_50_ = 25.45, 32.09 and 41.66 μg.mL^−1^, respectively), followed by *Aedes albopictus* (LD_50_ = 28.14, 34.09 and 44.77 μg.mL^−1^, respectively) and *Culex tritaeniorhynchus* (LD_50_ = 30.80, 36.75 and 48.17 μg.mL^−1^, respectively).

The essential oils of *Croton heliotropiifolius* Kunth. and *Croton pulegiodorus* Baill. were tested in larvicidal bioassays against *Ae. aegypti* L. [63]. *β*-caryophyllene was identified as the major compound in both essential oils (35.82% in *C. heliotropiifolius* and 20.96% in *C. pulegiodorus*). The essential oil of *C. pulegiodorus* (LD_50_ = 159 μg.mL^−1^) was more effective against *Ae. aegypti* than that of *C. heliotropiifolius* (LD_50_ = 544 μg.mL^−1^).

In order to verify whether *β*-caryophyllene is the active principle responsible for the observed larvicidal activity, this compound was purchased and its larvicidal potential was further evaluated herein. However, *β*-caryophyllene (LD_50_ = 1038 μg.mL^−1^) showed marginal larvicidal effectiveness against the *A. aquasalis* larvae. For this reason, the authors suggested a synergistic effect of components in the *P. nigrum* essential oil. The results of in loco larvicidal bioassays using the *in natura* essential oil is shown in Table 3.

An increased mortality as a function of the essential oil concentration (125, 250, 375, 454 and 625 µg.mL^−1^) was observed after 24 h (from 20% to 100%), which became less pronounced after 48 h (from 70% to 100%) and remained almost constant after 72 h (from 80% to 100%). Total mortality was obtained in all evaluated time (24 h, 48 h and 72 h) of exposure at concentration of 625 µg.mL^−1^. The LD_50_ and LD_90_ were calculated: after 24 h, values of 229.6 µg.mL^−1^ and 628.3 µg.mL^−1^ represented, respectively, the LD_50_ and LD_90_. After 48 h of exposure to the essential oil, these values decreased, respectively, to 166.9 µg.mL^−1^ and 370.2 µg.mL^−1^ and, finally, after 72 h they were found as 74.3 µg.mL^−1^ and 356.0 µg.mL^−1^. Considering the marginal variation in the number of deaths between 48 h and 72 h, a concentration slightly higher than that obtained after 48 h of exposure was considered for encapsulation (500 µg.mL^−1^).

The mechanism of action of essential oils in similar studies [64,65] reported a color change in the larvae corpuscle, according to the damage caused to their physiology. The coloring of the larvae through the different concentrations indicated damage to the neurological and digestive systems [66]. The change in the placement of the larvae head may be due to the inhibition of acetylcholinesterase (AChE), the enzyme responsible for the hydrolysis of the neurotransmitter acetylcholine (ACh) [66]. The larvae may have suffered the effect of the toxicity of the essential oil of *P. nigrum* by the combined action of their constituents. 

The bioassays using the biodefensive PNE also showed variation in mortality (considering the same concentrations) on *in vitro* and *in loco* bioassays, as shown in Table 4. From 100 μg.mL^−1^ to 200 μg.mL^−1^, larval mortality was higher on the *in loco* bioassay, probably due to the environmental conditions. Some parameters, such as humidity and controlled temperature [67], may have contributed to the particles’ destabilization, and consequently the release of essential oil. After 24 h, the mortality *in vitro* increased from (6.7 ± 0.8)% (100 μg.mL^−1^) to (37 ± 4)% (200 μg.mL^−1^), and after 48 h the mortality *in loco* increased from (8.3 ± 0.8)% (100 μg.mL^−1^) to (58 ± 4)% (200 μg.mL^−1^). The total larvae mortality on the *in loco* bioassays was almost reached (92%) after 24 h, and was completely reached (100%) after 48 h. The unloaded particles used as negative control (PUN) showed 0% mortality, as expected.

According to *in vitro* bioassays applying the *in natura* essential oil (Table 4), the concentration close to 500 μg.mL^−1^ (454 μg.mL^−1^) caused the death of 70% of the larvae after 24 h. Considering this same concentration (500 μg.mL^−1^) in the biodefensive PNE, the higher percentage of mortality observed (for both *in vitro* and *in loco* bioassays) may be related to the encapsulation of essential oil, delivering the loaded particles more efficiently inside the larvae, increasing the efficiency of the bioactive compounds. Similar results were also reported [18] considering a biodefensive based on PCL and *Lippia alba* essential oil. These results allowed important hypotheses on larvicidal effect. The authors suggested that the encapsulated particles may have penetrated the larvae cell membrane because the mortality time was considerably reduced (from 72 h for the *in natura* essential oil to 24 h for the loaded particles). Some authors also reported that most larvae ingest larger proportions of organic particulate matter up to around 50 μm [68], allowing the incorporation of bioactive compounds within several carriers and facilitating the ingestion process.

## 4. Conclusions

The present research successfully developed a controlled release system based on polymeric carriers for the encapsulation of the essential oil from *P. nigrum*. The developed biodefensives presented enhanced stability when maintained at 35 °C (according to the evaluated physical parameters) based on suitable preservatives to prolong their shelf-life storage. Through a controlled release study, the particles presented continuous release of essential oil at different pH, up to 72 h. Our results show that the biodefensive PNE is suitable for application at 35 °C, with a shelf-life of about 5 months. Bioactive compounds from medicinal plants are widely used by the population in general. In the encapsulated form, such molecules are greatly potentiated, despite the reduced concentration and the direct way they present themselves during the release process. Larvicidal bioassays against *A. aquasalis* using the biodefensive PNE showed mortality higher than 88% under *in vitro* and *in loco* conditions. One of the major challenges in designing and preparing functional materials is that their structural features need to be tailored toward the encapsulation and delivery of a specific bioactive compound. This perspective allows the development of polymer-based nanotechnology approaches for encapsulation and delivery of bioactive compounds. The usual management of mosquitoes may not be sufficient to effectively control malaria vectors, especially in Brazil, where urbanization promotes the proliferation of mosquitoes. Consequently, to achieve an effective vector control and substantially reduce the prevalence of malaria and other vector-borne diseases, an integrated management of these vectors must be adopted. In this perspective, the present study shows that a formulation based on *P. nigrum* essential oil may be taken into account in the integrated management of disease vector mosquitoes.

## Figures and Tables

**Table 1:** Average particle size of the biodefensive PNE measured by NTA.

Parameters	Biodefensive PNE
Mean (nm)	236 ± 34
Mode (nm)	136 ± 10
SD (nm)	157 ± 12
D_10_ (nm)	127 ± 10
D_50_ (nm)	178 ± 35
D_90_ (nm)	472 ± 78
Concentration (particles.mL^−1^)	(7.0 ± 0.5) × 10^10^

The parameters Mean, Mode, SD, D_10_, D_50_ (Median) and D_90_ indicate, respectively, the average, most frequent particle class size, standard deviation of the distribution, and the 10%, 50% and 90% percentiles of the analyzed particles.

**Table 2:** Coefficients of the Korsmeyer-Peppas mathematical model obtained from the controlled release of the biodefensive PNE.

Model	Coefficient	pH = 3	pH = 7	pH = 10
Korsmeyer-Peppas	k	92.7 ^a^	92.7 ^a^	89.8 ^b^
	n	0.64 ^a^	0.65 ^a^	0.64 ^a^
	R^2^	0.93	0.96	0.94

Different superscript lower-case letters (^a,b^) at the same column indicate significant differences (*p*-value > 0.05).

**Table 3:** The in loco LD_50_ and LD_90_ values of *Piper nigrum* essential oil against *A. aquasalis* larvae.

Time (h)	Concentration (μg.mL^−1^)	Mortality (%)	LD_50_ ± SD (μg.mL^−1^) LCL–UCL	LD_90_ ± SD (μg.mL^−1^) LCL–UCL	Equation
	125	20	230 ± 2 ^a^ (211.5–263.9)	628 ± 5 ^a^ (617.5–643.9)	Y = (−1.058 + 1.273) + 0.38X
	250	70			
24	375	60			
	454	70			
	625	100			
	NC	0			
	125	70	167 ± 2 ^b^ (152.6–181.2)	370 ± 3 ^b^ (366.8–378.2)	Y = (−0.826 + 1.610) + 0.44X
	250	90			
	375	90			
48	454	90			
	625	100			
	NC	0			
	125	80	74.3 ± 0.7 ^c^ (68.4–85.3)	356 ± 4 ^b^ (349.3–361.9)	Y = (0.243 + 0.818) + 0.41X
	250	90			
72	375	90			
	454	90			
	625	100			
	NC	0			

Results are expressed as mean ± standard deviations. Different superscript lower-case letters (^a–c^) at the same column indicate significant differences (*p*-value > 0.05). LD_50_: lethal dosage that kills 50% of exposed larvae, expressed in μg.mL^−1^; LD_90_: lethal dosage that kills 90% of exposed larvae, expressed in μg.mL^−1^; Y: mortality rate (significant at *p*-value < 0.05); X: concentration (significant at *p*-value < 0.05); SD, standard deviation; LCL: lower confidence limit; UCL: upper confidence limit. NC: Negative control.

**Table 4:** *In vitro* and *in loco* effectiveness of the biodefensive PNE on *A. aquasalis* larvae.

Time (h)	Concentration (μg.mL^−1^)	Mortality ± SD *in vitro* (%)	Concentration (μg.mL^−1^)	Mortality ± SD *in loco* (%)
	100	6.7 ± 0.8	100	8.3 ± 0.8
	200	37 ± 4	200	58 ± 4
24	400	88 ± 3	300	83 ± 6
	500	88 ± 1	500	92 ± 0
	NC	0	NC	0
	100	70 ± 2	100	10 ± 5
	200	82 ± 8	200	67 ± 2
48	400	92 ± 8	300	93 ± 6
	500	95 ± 5	500	100 ± 0
	NC	0	NC	0
	100	98 ± 3	100	25 ± 2
	200	97 ± 3	200	80 ± 5
72	400	97 ± 3	300	97 ± 6
	500	100 ± 0	500	100 ± 0
	NC	0	NC	0

NC: Negative control (unloaded particles—PUN system); SD: Standard Deviation.

## Data Availability

Not applicable.
